# Addressing the sustainability challenges for polymers in liquid formulations

**DOI:** 10.1039/d3sc90086j

**Published:** 2023-05-31

**Authors:** Caroline Louise Kelly

**Affiliations:** a CPI The Coxon Building John Walker Road Sedgefield TS21 3FE UK caroline.kelly@uk-cpi.com

## Abstract

Polymers in liquid formulations, or PLFs, are present in many of the products we use, from the shampoo we use to wash our hair, to the paint on the walls, and the lubricants in our car. They provide high functionality in these and a multitude of other applications, delivering many positive benefits to society. They are essential to global markets worth more than $1 trillion and so large quantities of these materials are made and sold each year – 36.3 million metric tonnes, the volume of 14 500 Olympic sized swimming pools! The chemical industry and the wider supply chain therefore have a responsibility to ensure that the way PLFs are made, used and disposed of at their end of life has a minimal effect on the environment. To date this seems to be a ‘hidden problem’, not receiving the same attention as other polymer related products, such as plastic packaging waste, yet there are clear challenges to address the sustainability concerns for these materials. To ensure that the PLF industry is economically and environmentally sustainable in the future, some key challenges need to be addressed, ensuring that new approaches to PLF production, use and end-of-life treatment are developed and utilised. Collaboration is key here, and with the UK already possessing a wealth of world-leading expertise and capability, there is an opportunity to leverage this in a coherent, focussed way to improve the overall environmental profile of these products.

## Abstract 2

Caroline Kelly introduces the sustainability challenges for polymers in liquid formulations and the role of chemistry in developing potential solutions to these sustainability challenges.

## Graphical abstract

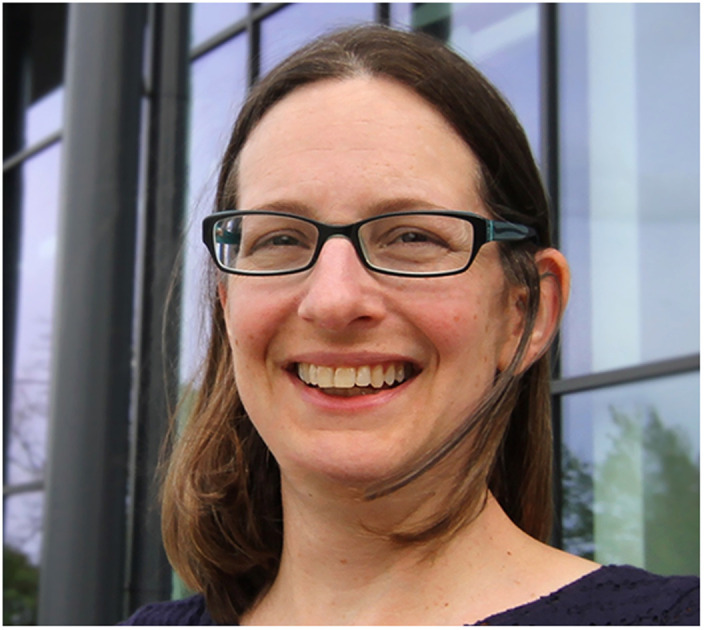

## Background

Now more than ever, people are aware of the need for ‘sustainable development’, *i.e.*, development which meets the needs of the present without compromising the ability of future generations to meet their own needs, as defined by the Brundtland Commission in 1987.^1^ The 2030 Agenda for Sustainable Development (https://sdgs.un.org/2030agenda), adopted by all United Nations Member States in 2015,^2^ sets out a blueprint to achieve this, and has at its heart 17 Sustainable Development Goals (SDGs), which are an urgent call for action by all countries – developed and developing – in a global partnership. This recognises that ending poverty must sit alongside strategies to improve health and education, reduce inequality, and spur economic growth – all while tackling climate change and working to preserve our oceans and forests.

The UK government is committed to delivering the 17 UN sustainable development goals. Encouragingly it was the first major economy in the world to pass laws to end its contribution to global warming by 2050 ^3^ and has set stretching targets around other environmental challenges, such as ending the sale of new petrol and diesel vehicles by 2030 ^4^ and banning single-use plastics in England by October 2023.^5^ However, reaching these targets will require extensive, systematic change and the chemical industry needs to be a key enabler of this.

Whilst society at large can see the need to remove plastic waste from the environment, and move to electrification, other areas of sustainability are less evident. Polymers in liquid formulations (PLFs) are one such challenge. These are widely used chemical products that provide a range of effects in formulation, *e.g.* rheology modification, emulsification, thickening, stabilisation, binding and film-forming. The chemical industry and the wider supply chain have a responsibility to ensure that the way PLFs are made, used and disposed of at their end of life has a minimal effect on the environment. On behalf of the Royal Society of Chemistry’s Synergy programme, which brings together businesses working in different industries to tackle complex chemistry subjects, and in consultation with industry, CPI collated a landscape view of the global PLFs market, and the sustainability challenges associated with them. The technical report^6^ produced from this work highlighted the significance of the role for chemistry in developing solutions and described some key opportunities to make PLFs more sustainable in the future.

## PLF market demand

The PLF market is technically diverse and complex, comprising hundreds of different polymer types within the categories of acrylic, epoxy resins, polyesters, polysilicones, polyurethanes, radiation curable, vinyl, water-soluble and other low volume polymers. They are produced from a variety of raw materials including natural, bio-based and fossil-derived monomers. PLFs are used widely across the Fast-Moving Consumer Goods (FMCG) sector, and due to the need for low cost and high availability combined with the properties they deliver in formulation the majority of PLFs manufactured are synthetic, *i.e.* the monomers are derived from petroleum oil and polymerised. As the global population grows, and particularly the increasing middle classes (*e.g.*, in China, India and Brazil),^7^ global demand for PLFs will increase, and with that the associated waste and negative environmental footprint. The Organisation for Economic Co-operation and Development (OECD) stated that the global consumption of materials will double by 2050 and annual waste generation will increase by 70%.^8^

## Sustainability challenges for PLFs

An analysis of the PLF landscape was conducted across eight key market sectors using these materials (adhesives and sealants, agriculture, household cleaning, inks and coatings, lubricants, paints and coatings, personal care and cosmetics and water treatment). This confirmed that these currently follow primarily a linear take–make–dispose model, with formulators selecting PLFs in the design of formulations primarily based on the specific properties and effects that they deliver rather than a consideration of sustainability. Yet a range of sustainability challenges exist for PLFs as discussed here and so it’s essential that this becomes a key factor considered in future product design.

### Reliance on fossil-derived feedstocks as raw materials

As discussed already, users of PLFs currently rely on synthetic PLFs for their products, but as demand increases for fossil-derived feedstocks, price increases, competition with other industries and increasing environmental strain create supply risks for the PLF industry. A solution is to move to natural polymers (*i.e.*, those occurring in nature which can be extracted or are produced by biological action), or polymers made from bio-based feedstocks (*i.e.*, where the monomers are produced from biological raw materials but prepared using chemical synthetic methods). Assuming that concerns around ethical or sustainable sourcing can be overcome, such sources also present a challenge in terms of greater variability in composition, presenting concerns over the consistency and robustness in manufacture.

### End-of-life waste

PLFs could be captured at the end of product life and re-used. However, this is a complex challenge; across the eight market sectors identified there are significant differences in the way that consumers and industry use and dispose of PLF products, with factors such as consumer behaviour and waste infrastructure strongly influencing the volume of PLF products consumed and their fate. For example, products such as shampoos and laundry detergents go straight down the drain after use and enter water treatment plants where they become diluted with a wide range of other compounds. Current biological treatment processes are unlikely to remove PLFs and therefore there is a high probability of them entering agricultural land as treated sludge; the PLFs are likely to be highly mobile in soil and water and therefore will spread widely and are effectively then acting as pollutants. Another example is PLFs in curable formulation systems such as paints and coatings; these may be difficult to remove from substrate materials at their end of life, meaning that materials such as composites, plastic packaging and traditional metal, wood and glass may not be readily-recyclable, and therefore end-up in landfill.

### Environmental pollution

PLFs in curable formulation systems such as paints and seed coatings may produce unintentional microplastics during use, *e.g.*, due to paint flaking or partial degradation of seed coatings and active ingredient delivery mechanisms. Uncontrolled release of these materials into the environment is likely to contribute to wider problems with microplastics pollution in marine and land environments.

## The role of chemistry in developing potential solutions to the sustainability challenge

There are three significant solution areas that could address the sustainability challenges of PLFs which are described below.

### Innovation in materials and formulation: biodegradable or naturally sourced alternatives

PLFs can become more sustainable by moving towards feedstocks from renewable sources, through the design of biodegradation for the end of their life and improving their efficiency in use so that fewer PLFs are needed in a formulation to deliver the same functional effects. To enable innovation in these areas, there are some key chemical science-based themes emerging as key areas for academia and industry to focus on to support the development of innovative yet sustainable ingredients and formulations.

### Novel biobased and biodegradable PLFs and natural alternatives

The use of biodegradable alternatives to existing PLFs is a potential route to sustainability. This would reduce the amount of fossil-derived feedstocks used and ensure that any waste generated has a positive effect on the environment, *e.g.* through soil regeneration. However, formulating with biodegradable ingredients presents a challenge in terms of ensuring they remain stable in the presence of other ingredients – formulations with high water content for example will catalyse degradation. The formulator therefore needs to balance this *vs.* the complete breakdown of PLFs into environmentally friendly products at the end of life, without compromising performance. One example is cellulose microparticles from the company Naturabeads; these would replace microplastics in rinse-off personal care products but could provide further insight into sustainable solutions for PLFs in paints and agrochemicals, which release microplastics into the environment throughout their lifetime.

There are promising examples of novel ‘natural’ alternative PLFs in the research literature. Examples for liquid formulations include chemically modified galactomannans as promising alternative to synthetic polyacrylamides,^9^ renewable polyacrylamide from naturally occurring terpene derivatives such as camphene from pine trees or waste from the paper industry^10^ and lubricants from biomass derived 2-alkylfurans and enals as an alternative to bio-ester based lubricants.^11^

A different approach is being pioneered by ViridiCO2; this company has developed a heterogeneous catalytic platform to use CO_2_ as a direct feedstock for high value chemicals, including polymers, and has the potential to reduce dependency on fossil fuel.

The challenge for PLFs in curable formulations is that they need to be durable to protect, join or seal substrate materials over long timeframes and so developing biodegradable alternatives may affect their long-term performance and durability. But also, since these PLFs are likely to remain on substrates at the end of their life, developing PLFs that degrade under controlled conditions or by triggering mechanisms could offer a way to effectively remove them in recycling processes so that substrate materials like glass, plastic and multicomponent materials can also be recycled. Environmental pollution occurring from uncontrolled release of microplastics from paint flaking and degradation of agrochemicals in the environment could also be eliminated, and PLFs entering landfill can be broken down into safe products. There are emerging novel solutions for further exploration, such as bio-based polyurethanes for agrochemicals and paints and coatings products from castor oil as alternatives to fossil-derived polymers for controlled release,^12^ citric acid and glycerol as replacements for waterborne polyurethanes^13^ and epoxidized soybean oil as UV curable waterborne polyurethane systems for use as pigment carriers in textiles^14^ as well as bio-based silicones from biogenic silica from rice hill ash.^15^ An interesting technology is that developed by Cambond; this is a low-cost bio-based resin that provides an environmentally friendly industrial adhesive to directly replace formaldehyde-based resins in products like MDF, particleboard and plywood. Cambond has also combined this resin with other biomass fibres or polymers to produce biocomposites, which can replace plastics in applications such as sustainable packaging, compostable materials and construction board manufacturing.

Against all this promise however there is still much work to be done, such as understanding the mechanisms by which PLFs biodegrade in the environment, their environmental fate and their potential breakdown products in different applications, sustainable sources of waste product for feedstock at industrial scale and low cost, bio-based PLF backbones that can be modified to match or exceed existing functionality and performance and biodegradable PLF backbones that break down completely under environmental conditions. Alongside this, developing triggered biodegradation mechanisms for key PLF backbones that have the potential to produce microplastics to reduce pollution is key, as well as developing PLF backbones that degrade under controlled conditions such as temperature, pressure and UV for recycling. There is also the challenge of feedstock variability and impact on final product performance; here there is an opportunity to develop digital twinning concepts, where product composition and manufacturing processes can be automatically varied in order to meet product specification despite changes in the feedstock.

### Improved PLF efficiency and performance

It may not always be possible to develop novel PLFs because of technical feasibility, cost or potential knock-on effects on formulations. However, there may be an opportunity to improve the efficiency and performance of formulations through innovation, which could improve sustainability by reducing either the amount of PLF required in a formulation, the product required in use, or the amount of other ingredients required in a formulation.

Some of the potential opportunities here include improving the durability of PLFs in paints to reduce the need for reapplication and reduce customer over-purchasing and increasing the bonding properties of PLFs in adhesives to reduce the amount of product that is required and the frequency of repair. Existing PLFs could be modified to create multifunctional materials that reduce the need for other ingredients in a formulation.

Some interesting examples of innovation to improve PLF efficiency and performance already exist. For example, mesoporous silica polydopamine composites derived from mussel proteins have been shown to improve the release of pesticide–fertiliser combinations^16^ and a waterborne siloxane modified polyurethane led to enhanced durability and almost doubled lifespan for paints and coatings.^17^ P&G have developed an alternative to the PLF carboxymethyl cellulose (CMC) called blocky carboxymethyl cellulose (BCMC) which makes fabric and dirt particle surfaces more negatively charged, so it is better able to repel soil particles from textiles once adsorbed onto cotton fabrics and soil particles, ultimately meaning less can be used in the laundry products.

### Platform technologies for formulation testing

The challenge for the formulator is that novel PLFs are likely to behave differently to existing polymers in a formulation, impacting stability, shelf life and performance. Additionally, PLFs are highly specialised materials with a wide range of functionalities, and therefore it is unrealistic for formulators to develop sustainable alternatives for every PLF in their product ranges. Understanding and ultimately predicting the behaviour of novel candidate PLFs and their effects on formulations would accelerate the development of innovative sustainable materials.

This is where predictive design enables a major step-change, and the use of tools such as machine learning-driven adaptive experimental design tools is a key enabler to develop statistical models to inform final product properties based on material inputs. Without this, trialling alternative feedstocks for PLFs or alternative PLFs in formulation comes down to stepwise trial and error in highly complex systems with multiple interacting ingredients. The use of automated experimentation can be built in here to generate large datasets for multiple compositions and then build models to understand and exploit the relationships between the ingredients, processing steps and resulting properties of the final formulation. Ultimately this type of approach can accelerate materials discovery, testing, and characterisation to dramatically reduce the time of materials development, yet this approach does not appear to have been widely adopted by the chemicals and materials sector. Some of the reasons for this have been summarised in a report by the Henry Royce Institute^18^ following engagement with representatives from academia, industry, and the High Value Manufacturing Catapult (HVMC) centres. It’s clear that there is a need for the development of integrated tools, infrastructure to meet the challenge, demonstrators and accelerators, and engagement, training and development of researchers and research leaders at the interface of science, engineering, and big data to provide the skills and training needed to drive this interdisciplinary transformation.

Clearly the use of high throughput/automated experimentation is only useful as a tool if relevant properties of the final formulation can be measured in a similarly accelerated timeframe. One of the key requirements for a formulated product is that it remains stable over its lifetime, which conventionally has been tested by leaving samples under a range of storage conditions and periodically assessing whether they are still stable or not. This is a real blocker to developing innovative formulations, and it’s for this reason that P&G and BP were prepared to co-invest in a project with CPI, funded by IUK through the National Formulation Centre grant, to develop tools to accelerate stability testing for liquid formulations.^19^ Combining this with automated formulation preparation and automated measurement of other key parameters, alongside robust data analysis would create a powerful tool for formulation development.

### Circular economy: re-using waste as new, valuable product

There is a clear opportunity to create a growing circular economy for PLFs in the market. For example, in the case of paints and coatings existing take-back schemes that reuse, repurpose and recycle paints could be scaled to a national level, reducing PLF waste and potentially feeding into alternative markets, such as the use of waste paint in construction, or the use of pyrolysis to recover TiO_2_ from waste paint. The fact that products such as adhesives, coatings, inks, paints and sealants are likely to have high PLF concentrations compared with that used in other industries may mean this is a more economically viable opportunity for this sector. The PaintCare^20^ initiative is a significant driver of activities in the UK, but there may be an opportunity to scale these up and expand to other markets such as adhesives and sealants. At scale, these could offer a circular solution for PLF products in curable formulation systems that remain unused pre-application. However, to achieve this will require significant effort and collaboration between UK governments, businesses and consumers.

Technologies could be developed to reclaim PLFs and reuse as secondary raw materials for the chemical industry, for example using hydrothermal separation and conversion technologies to convert PLFs in activated sludge and on substrate materials into high performance biomass to feed gasification processes, or the use of heat, solvent and hydrothermal separation and conversion technologies to reclaim monomers from PLFs on substrate materials for reuse. One example is Paraffinity; this company has developed a molecular binding technology to capture and remove target molecules from wastewater which could be applied to PLFs. The technology developed by Cambond described above is also advantageous in that it offers a fully circular solution that turns biomass by-products from agriculture into valuable materials available at scale.

The recently funded ‘Flue2Chem’ programme^21^ is an example of a ‘waste to worth’ programme engaging the full supply chain to develop solutions for capturing carbon dioxide from the flue gases from the paper and steel making sectors, turning this into a recognisable feedstock, synthesising this to make an alkoxyl surfactant and finally formulating this surfactant into cleaning products and coatings. Another example is where Nestle, Borealis, and their collaborators have utilised chemically recycled cross-linked polyethylene (PEX) waste to manufacture PEX pipes, which are commonly used in plumbing and heating applications. One of the partners, Wastewise, has developed an innovative pyrolysis-based chemical recycling to liquefy industrial waste PEX into an oil-like intermediate, which is co-processed by Nestle’s oil refinery to product a feedstock used by Borealis as a raw material to create the new pipe systems. The partners said that they were able to establish the value chain in little more than six months.^22^

Another opportunity is digital track and trace for paints and coatings and specific PLFs like polyacrylamide (PAM) which could improve industry’s understanding of supply risks and help develop more sustainable practices. It is widely recognised that adopting digital processes and automation can improve performance, which can cut costs, maximise resources and reduce waste.

### The potential role of biotechnology

Biotechnology uses the inbuilt biological processes of living organisms to take in substances and convert them into useful industrial products. It offers the possibility for a wide range of new ingredients derived from biological feedstocks and could be used to replace products derived from petrochemical feedstocks or made *via* chemical manufacturing processes. Global chemicals supplier Croda clearly sees biotechnology as an alternative route to more sustainable products, having recently produced two thought leadership pieces for the personal care and home care sectors.^23,24^

Ingredients produced by this route have the potential to deliver more consistent products without endangering biodiversity; for example, fermentation can be used to produce large amounts of a specific ingredient from a single microorganism rather than having to harvest huge amounts of crops to generate the same volume. Potentially this could offer a more sustainable route to novel monomers which could give improved performance when polymerised into PLFs and could mean lower levels of PLFs required to deliver the same benefits in formulation.

## Outlook

It’s clear that manufacturers, formulators, end users, and waste management companies are facing significant PLF sustainability challenges, with specific needs to reduce dependence on fossil-derived feedstocks, maximise resource efficiency and reduce waste generation in the long term. Any solutions to these challenges must be economically viable, available at industrial scale and accepted by the consumer, and as a minimum must match the performance of existing PLFs across a wide range of applications.

This presents a significant techno-commercial challenge where any single organisation is not able to take on the risk to solve. To drive innovation in this space, fill the existing knowledge gaps and exploit emerging technologies, a coordinated effort from players active across the ecosystem is required. Pre-competitive collaboration, common standards, standardised testing/life cycle analysis and infrastructure that supports and de-risks collaborative innovation are all essential components, as well as a common language. This is where the UK’s Catapult Network (with CPI being part of the High Value Manufacturing Catapult, and the centre most aligned with the chemical and materials science base) can be very useful in bringing together industry, academia and relevant supply chain partners, all of whom are enthused by the challenge and opportunity of collaboration but may not have the time nor capacity to take full ownership. Examples of where this has worked extremely successfully is through the National Formulation Centre strategic programme to develop new research infrastructure for the UK formulation industry, and the Medicines Manufacturing Innovation Centre’s ‘Grand Challenge’ approach to pre-competitive collaboration. As described above, the ‘Flue2Chem’ programme is an example of a programme engaging the full supply chain which is the approach required for PLFs.

Progressing any of the opportunities identified will require wide collaboration and significant long-term effort from academia, industry and the government – this is not the kind of thing which will happen spontaneously and without concerted effort. Encouragingly, with the RSC well placed to facilitate and coordinate this kind of collaboration, and with the PLF taskforce successfully up and running with representatives from key industry players, this is now well underway.

## Conflicts of interest

There are no conflicts to declare.

## Supplementary Material
